# Impact of prenatal and postnatal nutrition on pain modulation and stress response in offspring

**DOI:** 10.3389/fnut.2025.1657109

**Published:** 2025-10-16

**Authors:** Federica Cernigliaro, Vincenzo Raieli, Edvige Correnti, Giuseppe Santangelo, Rosaria Nardello

**Affiliations:** ^1^Department of Health Promotion, Mother and Child Care, Internal Medicine and Medical Specialities “G. D’Alessandro”, University of Palermo, Palermo, Italy; ^2^Department of Child Neuropsychiatry, ISMEP—ARNAS Civico–Di Cristina Benfratelli, Di Cristina Pediatric Hospital, Palermo, Italy

**Keywords:** prenatal nutrition, maternal nutrition, pain modulation, stress response, HPA axis, gut-brain axis, microbiota, inflammation

## Abstract

Nutrition is an environmental risk factor playing a pivotal role in predisposing to various diseases. Especially prenatal nutrition induces adaptation processes, known as early programming, leading to the alteration of fetal growth and brain development. Our previous study focused on the relation between prenatal nutritional factors and neurodevelopmental disorders. This narrative review analyses instead how prenatal and postnatal nutrition may impact many other pathways, for example, pain modulation and stress response. The alteration of these pathways is mediated by modification of the activity of the HPA (hypothalamic–pituitary–adrenal) axis, dysregulation of the gut-brain axis, and epigenetic changes induced by food. Diet and alterations in levels of macronutrients or micronutrients can alter the gene expression both in the uterus and early stage of life, increasing the susceptibility to many pathologies, related to metabolic alteration, but also cognitive impairment. Moreover, maternal diet can influence brain excitability and neuropeptides, and the release of neurotransmitters. We searched keywords such as “prenatal nutrition and pain modulation” on PubMed and Google Scholar, selecting the main reviews and excluding individual cases. Unfortunately, few data investigated this topic, so future perspectives may include more studies regarding the pathophysiology of these alterations, in order to understand how to improve and promote offspring’s health, through maternal and early nutrition.

## 1 Introduction

**Figure 1:**
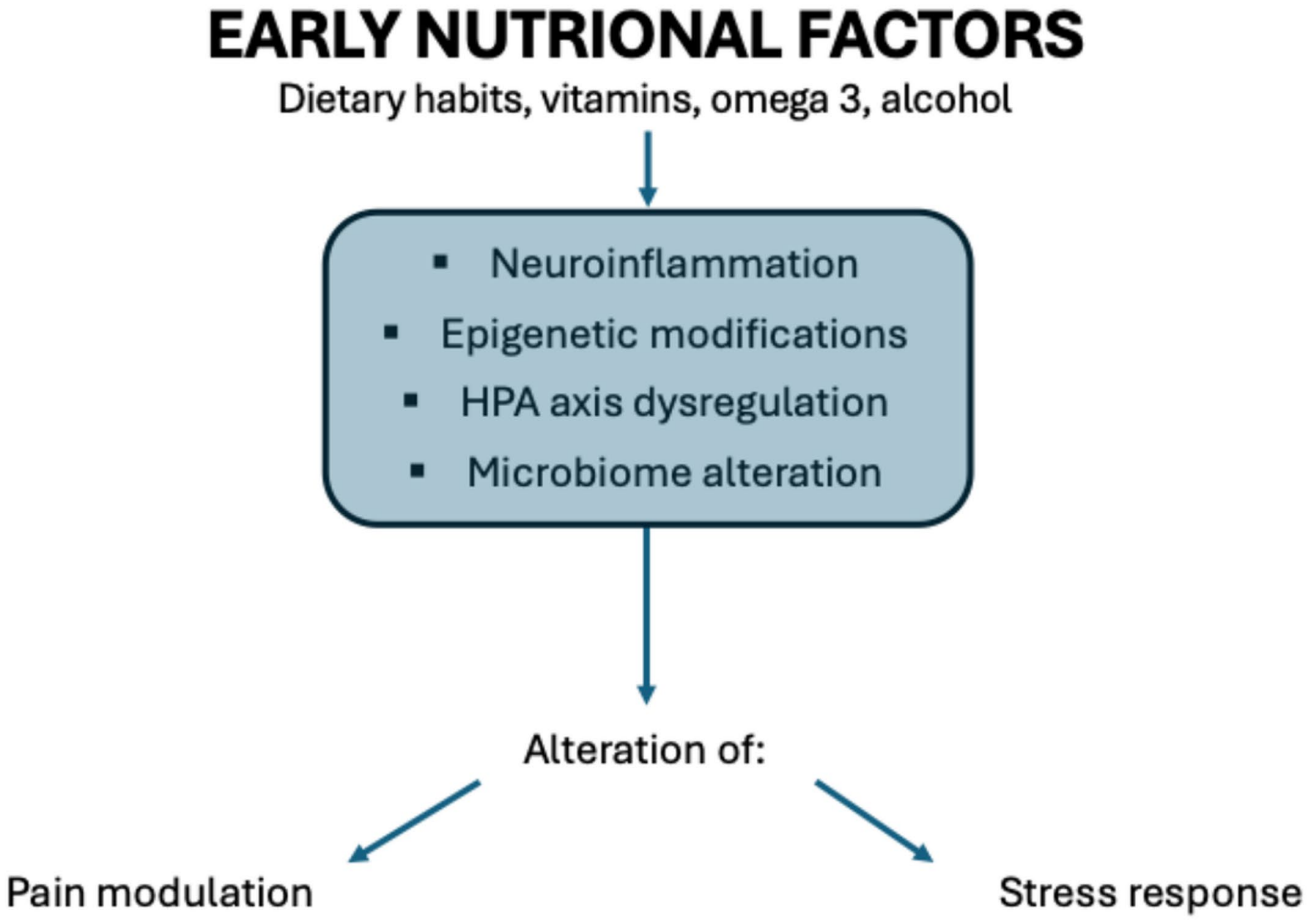
Correlation between nutritional factors and alteration in pain modulation and stress response. Illustration by Federica Cernigliaro.

Nutrition is an environmental risk factor playing a pivotal role in predisposing to various diseases. Especially prenatal nutrition induces adaptation processes, known as early programming, leading to the alteration of fetal growth and brain development. Our previous study (1) focused on the relation between prenatal nutritional factors and neurodevelopmental disorders. This narrative review analyses instead how prenatal and postnatal nutrition may impact many other pathways, for example, pain modulation and stress response. The alteration of these pathways is mediated by modification of the activity of the HPA (hypothalamic–pituitary–adrenal) axis, dysregulation of the gut-brain axis, and epigenetic changes induced by food (Figure 1).

So, early-life nutritional status can significantly influence the development of the nervous system, particularly affecting how individuals experience pain (nociception) and respond to stress throughout life. This concept aligns with the Developmental Origins of Health and Disease (DOHaD) framework, which emphasizes how early environmental factors, especially nutrition, can program physiological and behavioral responses (2).

## 2 Methods

We searched keywords such as “prenatal nutrition and pain modulation,” “prenatal factors and pain,” and “early nutrition and stress response” on PubMed and Google Scholar, selecting the main reviews from 2000 to 2025, excluding individual cases, so we identified 39 studies (Table 1).

**Table 1:** Summary of included studies.

Authors	Year	Study type	Main evidences
Delarue et al.	2003	Small-scale human study	Fish oil supplementation prevents adrenal activation from mental stress in healthy men
Bouret et al.	2004	Animal study (rodents)	Leptin exerts trophic effects on hypothalamic neurons regulating feeding
McMillen et al.	2004	Animal study (sheep)	Fetal nutrition influences prenatal leptin synthesis, programming risk for postnatal obesity
Palmer et al.	2004	Animal study (rodents)	Prenatal protein deprivation alters prepulse inhibition and NMDA receptor binding.
Walker	2005	Animal study (rodents)	Nutrition modulates neonatal brain development and stress responses.
Nieminen et al.	2006	Human study	Omega-3 fatty acids were associated with plasma neuroactive steroids in alcoholism and depression
Gatchel et al.	2007	Human study (review)	Biopsychosocial approach improves understanding of chronic pain mechanisms
Chrousus	2009	Human study (review)	Dysfunction of stress system contributes to psychiatric and endocrine disorders
Berti et al.	2011	Human study (review)	Micronutrient intake in pregnancy influences maternal and fetal outcomes; many unresolved questions remain
Soares et al.	2012	Animal study (rodents)	Maternal CLA diet enhances progeny growth and cortical spreading depression
Monk et al.	2013	Human study (review)	Prenatal stress and poor maternal nutrition interact to impair infant neurocognitive development
Mocking et al.	2013	Human study	HPA-axis dysregulation is linked to altered fatty acid metabolism in recurrent depression
Galland	2014	Human study (review)	Gut microbiome influences brain function and mental health
Prado & Dewey	2014	Human study (review)	Early-life nutrition is critical for optimal brain development
Sullivan et al.	2014	Animal study (rodents)	Maternal high-fat diet programs offspring behavior alterations
Debnath et al.	2015	Human study (review)	Fetal programming mechanisms contribute to schizophrenia risk
Godfrey et al.	2017	Human study (review)	Maternal obesity predisposes offspring to long-term metabolic and mental health risks
Menni et al.	2017	Small scale human study	Omega-3 fatty acids correlate with gut microbiome diversity in women
Gabbianelli and Damiani	2018	Human study (review)	Early-life nutrition exerts epigenetic influences on neurodegeneration risk.
Georgieff et al.	2018	Human study (review)	Nutritional adequacy is essential for structural and functional brain development
Maldonado-Ruiz et al.	2019	Animal study (rodents)	Maternal nutrition-induced inflammation modulates autism susceptibility
Li et al.	2019	Human study (systematic review)	Prenatal nutrition is linked to risk of neurodevelopmental disorders
Cryan et al.	2019	Human and animal study (review)	Gut microbiota–brain axis regulates stress, mood, and cognition
Dash et al.	2019	Human and animal study (review)	Gut microbiome influences brain development and neurodevelopmental disorders
Guo et al.	2019	Human and animal study (review)	Gut microbiota modulates pain regulation via immune and neural mechanisms
Franzago et al.	2020	Human study (review)	Gene–diet interactions influence chronic disease prevention in future generations
Raffaeli et al.	2021	Human study	Chronic pain terminology and implications in clinical practice
Gerasimova et al.	2021	Animal study (rodents)	Hyperhomocysteinemia increases cortical spreading depression and pain-related behaviors
Imado et al.	2022	Animal study (rodents)	Prenatal valproic acid exposure induces allodynia via spinal microglial activation
D’Ornelas et al.	2022	Animal study (rodents)	Fish oil supplementation reduces inflammatory pain short- and long-term
Cömert et al.	2022	Small-scale human study	Pre-pregnancy obesity alters maternal and fetal microbiome, linked to fetal growth
Bekdash et al.	2024	Human study (review)	Epigenetics and nutrition interact in brain health and mental illness risk
Cernigliaro et al.	2024	Human study (review)	Prenatal nutrition is a key factor in neurodevelopmental disorder risk
Halloum et al.	2024	Human study (systematic review)	GLP-1 receptor agonists show promise for headache and pain disorders
Kodila et al.	2024	Animal and human study (review)	Perinatal trauma alters epigenetic regulation and chronic pain risk
Król et al.	2024	Animal study (rodents)	Prenatal alcohol exposure modifies nociceptive responses in offspring
Zinkow et al.	2024	Animal and human study (review)	Omega-3 fatty acids influence gut–brain axis via molecular mechanisms
Exposto et al.	2025	Human study	Prenatal nutrition increases risk of temporomandibular disorders and headaches in young adults
Sgro et al.	2025	Animal study (rodents)	Perinatal trauma alters nociception and gene expression in adolescent rat brains

## 3 Pain modulation

According to the International Association for the Study of Pain (IASP), “Pain is an unpleasant sensory and emotional experience associated with, or resembling that associated with, actual or potential tissue damage” (3). This definition underlines that pain is not purely a physical phenomenon; it also involves emotional and cognitive components. Pain is a subjective experience that is valid and real, even in the absence of visible tissue damage.

The pathophysiology of pain involves the peripheral and central nervous systems, along with chemical mediators, receptors, and neural pathways. It consists of four phases (4):

1. Transduction, during which a noxious stimulus (mechanical, thermal, or chemical) is converted into an electrical signal by nociceptors (pain receptors) and activates free nerve endings in the skin, muscles, joints, and internal organs. It involves inflammatory mediators, such as bradykinin, prostaglandins, histamine, serotonin, and substance P, that sensitize nociceptors.
2. Transmission, during which the nociceptive impulse travels along afferent nerve fibers up to the dorsal horn of the spinal cord, conneting with interneurons and is transmitted upward to the central nervous system (CNS) through the spinothalamic tracts. Aδ fibers, myelinated, are responsible for fast conduction of sharp and localized pain. C fibers, unmyelinated, are responsible for slow conduction of dull and persistent pain.
3. Perception, which is the conscious awareness of pain, is processed by several brain areas: the somatosensory cortex for localization and intensity; the limbic system for the emotional component of pain; the prefrontal cortex for cognitive evaluation and behavioral response.
4. Modulation, during which the CNS can inhibit or amplify the pain signal through descending pathways originating from the medulla, periaqueductal gray (PAG), and raphe nuclei. Different neurotransmitters are involved, such as endorphins, enkephalins, serotonin, and norepinephrine. These systems regulate pain intensity and form the basis for the action of opioids and certain antidepressants.

There are different types of pain (5):

- Nociceptive pain, evoked by activation of nociceptors and based on the site of onset, is divided into superficial somatic (coming from the skin), deep somatic (coming from muscles, bones, joints, and connective tissue), and visceral (coming from the internal organs). Moreover, it can be well-localized or dull;
- Neuropathic pain, produced by damage at different levels of the central and peripheral nervous system;
- Inflammatory pain, produced by the tissues within our body as a reaction to the harmful stimuli in order to eradicate the necrotic cells and initiate the tissue repairing process. Inflammation may lead to three major responses: hyperalgesia, allodynia and sympathetic maintained pain.

So, pain is not just a symptom but a complex physiological process, modulated by biological, psychological, and social factors.

## 4 Stress response and HPA Axis

The stress response is the body’s coordinated physiological and behavioral reaction to a perceived threat or challenge (a stressor). Its main purpose is to help the organism restore homeostasis and prepare for “fight-or-flight.”

Stress response is mediated by two components (6):

- Sympathetic-adrenal-medullary (SAM) axis, involved in fast-acting, releasing adrenaline and noradrenaline;
- Hypothalamic–pituitary–adrenal (HPA) axis, slower, but with longer-lasting effects through release of glucocorticoids (mainly cortisol).

HPA axis is a central neuroendocrine system that regulates the stress response and many fundamental physiological functions (7). It involves three main components:

- Hypothalamus, which detects stress signals from the brain (e.g., amygdala, hippocampus) and releases corticotropin-releasing hormone (CRH).
- Pituitary Gland, which, in response to CRH, releases adrenocorticotropic hormone (ACTH) into the bloodstream.
- Adrenal Cortex, stimulated by ACTH to produce and release cortisol.

In case of chronic stress, the HPA axis may become dysregulated, leading to constantly high or blunted cortisol levels and increased risk for depression, anxiety, immune dysfunction, metabolic disorders, and altered pain sensitivity. HPA overactivation leads to Cushing’s Syndrome, with cortisol excess, and HPA underactivation leads to Addison’s disease, with cortisol deficiency.

In children, adverse early experiences (e.g., poor nutrition or trauma) can permanently alter HPA axis function.

Cortisol, known as the stress hormone, is involved in the “fight or flight” response to stressful situations, increasing the energy available to deal with the threat. It also plays several roles: it regulates macronutrient metabolism, ensuring the body has the right energy; it has anti-inflammatory effects, balances the sleep–wake cycle and regulates the blood pressure; it increases glycemia for quick energy and suppresses non-essential functions (e.g., digestion, growth, reproduction); it suppresses immune system activity and influences memory, mood, and cognition.

After the stressor is gone, negative feedback mechanisms reduce CRH and ACTH release, lowering cortisol levels and restoring balance.

## 5 Early factors influencing nociception

Different early factors can impact nociception, for example, perinatal trauma, drugs, and nutrition.

The prenatal and postnatal period represents a critical phase for neurodevelopment, in which the brain is highly susceptible to various noxae.

A recent study (8) investigated the relation between perinatal trauma and alteration of nociception, showing that trauma, such as intimate partner violence and early life stress/neglect during the perinatal phase, increases the risk for chronic pain. It leads to immune dysregulation, dysfunction in stress-reactivity, microglia activation, and transcriptomic changes in the prefrontal cortex and hypothalamus of adolescent rats, modifying offspring behavior and nociceptor sensitivity. So, perinatal trauma impacts cognitive, socio-emotional, and pain-processing in offspring, with changes in gene expression, in both mothers and offspring.

Epigenetics focuses on the molecular processes modulating gene expression without changing the DNA sequence, such as DNA methylation, modification of histones, and regulation of microRNAs (miRNAs).

Another study (9) also focused on critical windows, analysing the association between perinatal trauma and epigenetics and chronic pain. They demonstrated that trauma during perinatal and early postnatal years (such as intimate partner violence while in utero or adverse childhood experiences) can determine epigenetic modification leading to neural reorganization and central sensitization, increasing the risk of pain hypersensitivity and chronification of pain. This study also suggests prophylactic strategies, such as oxytocin administration and probiotic use, to attenuate the epigenetic modifications induced by early exposure to trauma.

Drugs may also impact pain processing. Another study showed that prenatal valproic acid treatment causes allodynia, associated with spinal microglial activation, leading to increased nociceptive responses (10).

## 6 Nutritional factors

Prenatal and postnatal nutrition is essential for fetal development.

In our previous study we have extensively discussed the importance of preventive nutrition, underlying the central role of food in the prevention of many chronic diseases: eating habits, such as high consumption of fats and sugars, street food, alcohol abuse, and reduced intake of fruit and vegetables, constitute risk factors for heart disease, cancer, diabetes, and various other pathologies (11).

Diet and alterations in levels of macronutrients or micronutrients can alter the gene expression both in the uterus and early stage of life, increasing the susceptibility to many pathologies, related to metabolic alteration, but also cognitive impairment. Moreover, maternal diet can influence brain excitability and the release of neuropeptides and neurotransmitters.

So, exposure to an unbalanced diet in early life and during the life span negatively modulates gene expression, leading to epigenetic changes associated with alteration of different neural pathways, neurodevelopmental disorders, and neurodegenerative diseases later in life (12).

### 6.1 Main mechanisms involved

Several mechanisms are involved in the correlation between nutrition and modulation of pain and stress response, especially neuroinflammation, epigenetic modifications, HPA axis dysregulation, and microbial dysbiosis.

Inadequate dietary pattern (pro-inflammatory foods and diets) determines a chronic systemic inflammatory state, leading to neuroinflammation, central sensitization, and impaired neuroendocrine regulation. Moreover, a pro-inflammatory diet can also determine HPA axis dysregulation, leading to elevated levels of cortisol, leaky gut, systemic inflammation, and neuroinflammation. Modification of this axis activity can cause alteration of GLP-1, a hormone secreted by the gut, involved in pain modulation through reduction of neuroinflammation thanks to the increase of anti-inflammatory mediators such as IL-10 and *β*-endorphins (13).

Nutrients can determine epigenetic modifications (changes in gene expression without altering DNA sequence), acting as methyl donors (e.g., folate, choline), modifying gene expression, influencing pathways related to stress and nociception: food can lead to epigenetic regulation through DNA methylation (11); in fact, nutritional experiences in the first years of life or maternal nutrition both in prenatal and postnatal phases can lead to epigenetic changes, inducing persistent metabolic and physiological changes, increasing the susceptibility to various chronic diseases in later stages of life.

Moreover, diet plays an important role in the development and modification of microbiota, also through epigenetic mechanisms. Microbiome alterations include reduced microbial diversity (especially depletion of anti-inflammatory taxa, such as *Faecalibacterium prausnitzii* and *Coprococcus comes*), alteration of neurotransmitter signalling (GABA and serotonin) by dysbiosis, alteration of short-chain fatty acids (SCFAs, especially butyrate, propionate, and acetate) produced by gut microbiota, and leaky gut. These conditions are related to neuroinflammation.

Different studies have also shown that the early shaping of the microbiome plays a crucial role in microglial maturation and modulates glial activation in the central nervous system. This modulation is considered a key factor in regulating neuroinflammation within the CNS (14).

The brain-gut axis (BGA) consists of a bidirectional communication between the central nervous system and the gastrointestinal system, also through the vagus nerve, involving neuronal, immune, endocrine, and metabolic. The interplay between brain and gut (also called the “second brain”) influences peripheral and central sensitization, playing an important role in the pathophysiology of chronic pain.

Chronic pain is defined as pain persisting or recurring for longer than 3 months (15). It is a long-term condition associated with significant emotional distress or functional disability and cannot be better explained by another condition, according to the International Association Study of Pain classification (16). Chronic pain is influenced both by central nervous system mechanisms and psychosocial factors (17), and its management requires a multidisciplinary approach, including pharmacological treatments, psychological support, physical rehabilitation, and lifestyle modifications.

The CNS also communicates with the gastrointestinal tract through the HPA axis, a key component of the gut–brain communication (18). Dysregulation of this system, particularly HPA axis hyperactivity, is implicated in the pathophysiology of anxiety, depression, and other stress-related conditions (19). Moreover, brain-gut axis dysregulation seems to be related to chronic pain conditions such as headache and fibromyalgia (20), through mechanisms related to microbial diversity, inflammation, and gut barrier integrity, but also neuroendocrine modulation, autonomic nervous system (ANS) involvement, and HPA axis dysregulation.

### 6.2 Prenatal nutrition

During gestation, the fetus undergoes critical phases of development.

Adequate maternal nutrition is essential for the optimal formation of neural circuits involved in the hypothalamic–pituitary–adrenal (HPA) axis and the endogenous pain control system, including opioid and cannabinoid pathways.

Maternal nutrition can modulate gene expression through epigenetic mechanisms; for example, the methyl-donor micronutrients of the one-carbon metabolism, such as folic acid, vitamin B6 and B12, and choline, play important roles in many physiological pathways and processes, including DNA methylation (21).

As regard nutrients, omega-3 fatty acids, folate, vitamin D, vitamin B12, vitamin B6, and choline may impact pain modulation and stress response through different mechanisms.

Deficiencies or imbalances in key nutrients such as omega-3 fatty acids, B vitamins, magnesium, and protein during pregnancy can lead to:

- Alteration of the development of pain-processing centers in the brain and spinal cord;
- Increased sensitivity to pain (hyperalgesia) in later life;
- HPA axis dysregulation, which controls cortisol release and stress adaptation.

Dietary pattern influences gene expression too: maternal undernutrition or high-fat diets have been associated with epigenetic modifications that predispose offspring to increased stress reactivity and impaired pain inhibition mechanisms.

Table 2 summarizes the role of the main nutritional factors according to the data cited below.

**Table 2:** Interaction between the main nutritional factors and pain modulation.

Nutritional factors	Mechanisms involved
Maternal undernutrition	↑ activity of the HPA axis (27)
Maternal HFD	MIA (maternal immune activation) and chronic inflammation (23, 35)
Maternal obesity	↑ activity of the HPA axis (28) Chronic inflammation and neuroendocrine alterations (28) Alteration of dopamine and serotonin pathways through epigenetic (28) Alteration of microbiota (29)
Omega-3 fatty acids	Neurotransmitter function, signal transduction, neurogenesis, synaptogenesis, and myelination (1) Influence on the gut microbiota and modulation of the gut-brain axis (31) Regulation of the HPA axis (31–34) Anti-inflammatory properties (31)
Folates	Metabolic pathways, neurogenesis, neuronal proliferation, migration, differentiation, vesicular transport, apoptosis, synaptogenesis, and myelination (1) DNA methylation (1) Prevention of neural tube defects (37)
Vitamin D	Regulation of gene expression (27) Neuronal differentiation, neurotransmission, calcium homeostasis (27)
Vitamin B12	Myelination, metabolic pathways, haemoglobin synthesis (38) DNA methylation (48) Prevention of neural tube defects (37)
Vitamin B6	Metabolic pathways (21) Development and maintenance of CNS and immune system (21) DNA methylation (21)
Choline	Metabolic pathway (39) DNA methylation (39) Neurogenesis and angiogenesis in the fetal hippocampus (27)
Chronic alcohol exposure	↓ sensitivity to nociceptive stimuli (25)

#### 6.2.1 Evidence from animal studies

Several preclinical studies provide mechanistic insights into how prenatal nutrition affects neurodevelopment, pain modulation, and stress response.

Sullivan et al. (22) demonstrated that maternal high-fat diet (HFD) in rodents increased inflammatory cytokines and altered brain reward circuits (nucleus accumbens, ventral tegmental area, prefrontal cortex) through hypomethylation of opioid gene promoters, predisposing offspring to obesity and stress dysregulation. Limitations of this study regards animal models, which may not fully capture the complexity of human diets and genetic backgrounds, so the results cannot be directly extrapolated to humans.

Maldonado-Ruiz et al. (23) showed that in mice, HFD-induced maternal immune activation (diet-induced MIA) determines a chronic pro-inflammatory profile during fetal development, leading to hyperactivation of microglia The cross-talk between the immune system and CNS induces epigenetic modifications on immune cells in offspring, such as DNA hypermethylation and aberrant expression of proinflammatory genes in the CNS, responsible for micro- and macrostructural defects in several brain regions, leading to neurodevelopmental abnormalities. But rodent immune responses differ from humans, so these findings require validation in clinical studies.

D’Ornelas et al. (24) found that in rats, fish oil supplementation reduced inflammatory pain either when consumed during adult life or during prenatal development. Unfortunately, controlled dietary conditions in animal models may not reflect real-world human nutritional variability.

Król et al. (25) demonstrated that chronic prenatal alcohol exposure (PAE) in rats reduced nociceptive sensitivity to mechanical and thermal stimuli in early postnatal life. But alcohol exposure patterns in animals may not replicate human drinking behaviors during pregnancy.

Gerasimova et al. (26) showed that in rats, hyperhomocysteinemia increased susceptibility to cortical spreading depression associated with photophobia, mechanical allodynia, and anxiety-related behaviors. But elevated homocysteine was experimentally induced, so translational relevance to naturally occurring human metabolic disorders remains uncertain.

These animal studies highlight potential mechanisms (epigenetic reprogramming, inflammatory pathways, neurotransmitter regulation), but their external validity is limited by species differences and controlled experimental conditions.

#### 6.2.2 Evidence from human studies

Human studies and reviews provide epidemiological and mechanistic evidence on the role of maternal nutrition in prenatal neurodevelopment and pain regulation.

Bekdash (21) realized a review emphasizing how maternal intake of methyl-donor micronutrients (folic acid, B6, B12, choline) influences epigenetic regulation and brain development. Especially about vitamin B6, which plays an important role as a coenzyme in the metabolism of amino acids, carbohydrates, and fats, and it is important for the correct development and maintenance of the CNS and immune system. Moreover, it is essential for serotonin synthesis and is involved in the regulation of mood, sleep, appetite, memory, and concentration skills. It plays a key role in regulating methylation mechanisms, influencing gene expression, and various pathways involved in neurodevelopment and neurological functions. This study, as a narrative review, relies on secondary data without new experimental evidence.

Debnath et al. (27) wrote a review on fetal programming of schizophrenia, analysing the link between maternal undernutrition and HPA axis disregulation and glucocorticoid receptor expression: maternal undernutrition seems to determine epigenetic changes in the fetal brain related to an increased expression of genes coding for the glucocorticoid and proopiomelanocortin receptor at the level of the fetal hypothalamus, leading to increased activity of the HPA axis. Moreover, this study analysed how vitamin D can regulate gene expression, influencing brain development. Currently, the recommended intake during pregnancy is 600 UL/day. Prenatal vitamin D deficiency causes brain development abnormalities and alterations of neuronal differentiation, neurotransmission, various pathways, and calcium homeostasis, as well as post-translational modifications, underscoring the role of this vitamin in epigenetic regulation. Choline also has an important role in neurodevelopment, being implicated in adaptive modulation of cognitive functions. Therefore, maternal choline deficiency alters neurogenesis and angiogenesis in the fetal hippocampus. In fact, according to some studies, oral integration of choline in the second trimester and after birth seems to be associated with better sensorial gating. Limitations of this review regards the mechanistic hypotheses largely based on animal data and indirect human evidence.

Godfrey et al. (28) showed that maternal obesity during pregnancy could induce immune-inflammatory modifications, altering fetal neuronal pathways involved in neurodevelopment, leading to alteration of HPA activity, and epigenetic changes of neurotransmitter pathway in offspring. Among the various mechanisms, there is an increased expression of glucocorticoid receptors at the hippocampal level with changes in HPA axis activity and an increased expression of fetal cytokines, such as IL-6, leading to inflammation and neuroendocrine alterations. Moreover, epigenetic changes intervene in the dopamine and serotonin pathways. This review is based on observational human data, so causality is difficult to establish.

Cömert et al. (29) showed, through a clinical study, that pregnant obesity can also affect a newborn’s microbiota. Meconium samples of infants born to mothers who were obese pre-pregnancy showed an altered microbiota, with fewer Firmicutes and increased Proteobacteria, compared with those of infants born to mothers with normal pre-pregnancy BMI. Limitations of this article is the small-scale study; moreover, microbiome is influenced by multiple postnatal factors.

Exposto et al. (30) analysed pre-natal nutrition as risk factor for painful temporomandibular disorders and headaches in young adults: they conducted a prospective longitudinal cohort study, analysing survey data from the Danish National Birth Cohort (DNBC), including prenatal nutritional information assessed using a healthy eating index (HEI) derived from a food frequency questionnaire collected between 1996 and 2002 and offspring’s p-TMD and headache status at age 18–23 years, assessed in 2021. In conclusion, they found that offspring of mothers with higher HEI scores, corresponding to healthier dietary choices during pregnancy, had significantly lower odds of headaches, but they did not find a significant link with painful temporomandibular disorders in young adulthood. Lower maternal intake of saturated fatty acids and red meat were associated with decreased odds of reporting headaches later in life. It’s an observational design, in which self-reported dietary intake is subject to recall bias; moreover, it cannot infer causality.

Our previous study (1) is a review highlighting the roles of the main nutrients (omega-3 s, folate, vitamin D, B6, B12, and choline), but also dietary patterns, in neurodevelopment, analysing the association between prenatal nutritional factors and neurodevelopmental disorders. We studied the role of omega-3 fatty acids in the CNS, as components of neuronal membrane and of myelin sheet, influencing neurotransmitter function, signal transduction, but also neurogenesis, synaptogenesis, and myelination, improving cognitive functions. Moreover, folates play a role in many metabolic reactions and pathways, but also neurogenesis, neuronal proliferation, migration, differentiation, vesicular transport, apoptosis, synaptogenesis, and myelination. It’s a review, that relies on secondary data without new experimental evidence.

Zinkow et al. (31) produced a review describing how omega-3 fatty acids modulate the gut–brain axis, HPA axis activity, and inflammation. Especially EPA and DHA, have been shown to regulate the HPA axis by reducing excessive cortisol production, associated with stress responses and mental health disorders. Omega-3 fatty acids have anti-inflammatory properties by altering eicosanoid production, reducing proinflammatory cytokines, and promoting anti-inflammatory mediators. Additionally, omega-3 fatty acids, particularly DHA, can influence the composition and function of the gut microbiota, promoting beneficial bacterial populations’ abundance and gut microbiome diversity, which contribute to gut health and improve systemic immunity. At the same time, gut microbiota can affect the absorption and metabolism of these fatty acids. Fish oil, for instance, reduces the growth of Enterobacteria while increasing that of Bifidobacteria. These actions help preserve the integrity of cellular barriers like the intestinal and blood–brain barriers. This review integrates preclinical and clinical data, but specific causal pathways remain unclear in humans.

Delarue et al. (32) showed, through a small human trial, that fish oil prevents stress-induced adrenal activation: they found that low plasma levels of omega-3 fatty acids are correlated with higher CRH and plasma concentration of cortisol, while omega-3 fatty acid supplementation may decrease CRH expression and corticosterone secretion. Unfortunately, it’s about a limited sample, with short-term intervention and an uncertain generalizability to pregnant populations.

Mocking et al. (33) demonstrated, through a clinical study, the link between HPA-axis dysregulation and altered fatty acid metabolism in recurrent depression, showing that omega-3 fatty acid supplementation may decrease CRH expression and corticosterone secretion. It’s an observational design and cannot establish directionality.

Nieminen et al. (34) found that omega-3 fatty acids correlate with plasma neuroactive steroids in patients with alcoholism and depression: particularly, excessive stress response and HPA hyperactivity may be linked to higher concentrations of brain and plasma neuroactive steroids (NASs), and be related to lower plasma levels of omega-3 fatty acids This study is based on small clinical cohort and comorbidities may confound associations.

Menni et al. (35) showed, through an observational study in women, that omega-3 intake correlates with gut microbiome diversity: particularly DHA, can influence the composition and function of the gut microbiota, promoting beneficial bacterial populations’ abundance and gut microbiome diversity, which contribute to gut health and improve systemic immunity. But it’s about a cross-sectional design and causality cannot be established.

Li et al. (36) produced a systematic review showing the association between prenatal nutrition and neurodevelopmental disorders. Especially, they highlighted that a decreased folate intake provokes incorrect DNA methylation, leading to an alteration of brain development. Limitations are related to heterogeneity of included studies; moreover, some data is based on self-reported dietary intake.

Berti et al. (37) highlighted, through a review, the importance of micronutrient supplementation in pregnancy, recommending periconceptional and prenatal supplementation of folic acid in the prevention of neural tube defects, reminding that data recommend a periconceptional supplementation of 400 mg/day. Moreover, they found an association between deficiency of vitamin B12 during pregnancy (values below 200 pg./mL) and irritability, reduced brain growth, and increased risk of neural tube defects. Unfortunately, some recommendations derived from population-level data, with evidence gaps.

Georgieff et al. (38) emphasized, through a review, the importance of vitamin B12 and other nutrients for brain myelination and development. Particularly, vitamin B12 acts as an enzyme and as a cofactor and is essential for the metabolism of fats and proteins, for haemoglobin synthesis, and contributes to DNA methylation. This review is mainly a conceptual synthesis, with limited longitudinal human trials.

Prado and Dewey (39), analysed choline’s role in neurodevelopment and epigenetic modulation. Choline plays a role in various pathways, including the synthesis of phospholipids and neurotransmitters. It acts as a methyl group donor, leading to epigenetic modifications in the fetal brain and placenta through mechanisms of DNA methylation; moreover, it is involved in the proliferation of stem cells and transmembrane signalling during neurogenesis. This review is based primarily on preclinical evidence, with scarce human intervention studies.

Together, human studies support the importance of balanced maternal nutrition (especially omega-3 fatty acids, B vitamins, choline, and vitamin D) in regulating stress response, pain sensitivity, and neurodevelopment, though limitations (observational designs, self-reporting, and heterogeneous methods) restrict firm causal conclusions.

### 6.3 Postnatal nutrition

The early postnatal period, especially the first 1,000 days of life, continues to be a sensitive window for neuroplasticity. Breastfeeding has been linked to better neurodevelopmental outcomes, partly due to its content of long-chain polyunsaturated fatty acids (LCPUFAs) and bioactive peptides, which support the maturation of brain regions involved in emotion regulation and sensory processing. DHA present in breast milk plays a pivotal role for the correct brain development, also influencing regulation of HPA axis and microbiota, through mechanisms described above.

Early exposure to ultra-processed foods, sugar excess, unhealthy dietary patterns (“Western” diet), or alteration of micronutrients may determine:

- Impairment of neurotransmitter synthesis (e.g., serotonin and dopamine).
- Disruption of gut microbiota composition, which influences brain-gut axis signalling.
- Weakening of stress resilience and heightened vulnerability to chronic pain syndromes and pain perception.

Postnatal nutrition shares the same mechanisms and the same consequences as maternal (prenatal) nutrition.

The lactation seems to be the most critical period for diet interventions.

#### 6.3.1 Evidence from animal studies

Palmer et al. (40) investigated food restriction during lactation in rodents, showing alteration in brain structures such as hippocampus, striatum, hypothalamus, and cortex, which are critical areas for external information processing and stress responses. This study is conducted in rodents, thus results may not fully translate to humans. Moreover, the restricted diet model may not represent the complexity of human nutritional deficiencies.

McMillen et al. (41) reported that maternal overnutrition during lactation in sheep can predispose offspring to altered energy balance and metabolic syndrome. This study focused on sheep, which may not perfectly replicate human metabolic development; moreover, long-term neurobehavioral effects were not extensively studied.

Bouret et al. (42) found that postnatal overfeeding in rodents can affect synaptic plasticity of specific neuronal pathways involved in metabolic regulation and stress response. Unfortunately, rodent neurodevelopment occurs at a different pace compared to humans, limiting extrapolation.

Walker (43) explored how macronutrients (carbohydrates, fats, proteins) affect neonatal brain development and stress responses in rodents, reporting that sucrose ingestion reduced stress and pain responses, while fats influenced HPA axis regulation and neuroplasticity. Carbohydrate ingestion affects several neuropeptides and neurotransmitter system, including neuropeptide Y (NPY), serotonin, dopamine, norepinephrine and the release of endogenous opioids (EOP). Increased carbohydrate intake might subserve functions related to coping with pain and making cognitive memories. Indeed, sucrose has often been associated with opiate-like effects in the brain. Sucrose ingestion induces changes in several neuropeptide and neurotransmitters systems in the CNS mainly via the activation of vagal afferents and other afferents to the nucleus of the tractus solitarius (NTS) and catecholaminergic cell groups in the medulla and locus coeruleus. Inputs are relayed to regions that are critical for the regulation of the HPA axis such as the paraventricular nucleus (PVN), amygdala and BNST. Some of the resulting effects of sucrose ingestion include a reduction in stress responses and pain and an increase in cognitive appraisal and reward value of the stimuli associated with sucrose ingestion. Also, fat ingestion plays a role in regulation of the HPA axis and stress responses. The production of free fatty acids (FFA) from triglycerides affects several organs including a stimulation or inhibition of the adrenal glucocorticoid production in rodents and humans, respectively. In rodents, FFA are suggested to stimulate CRF production. FFA also affect liver production of corticosterone binding globulin (CBG), insulin secretion from the pancreas and adipose tissue growth and release of leptin, cytokines, etc. Leptin has been shown to inhibit adrenal glucocorticoid production and hypothalamic CRF synthesis, while cytokines such as IL-1beta have the opposite effect to stimulate the HPA axis. Fat can also alter the expression of several hypothalamic neuropeptides that impact on CRF synthesis and secretion. The consequences of feeding on a high fat diet on neuroplasticity and cognition are thought to be mediated by changes in free radical production, and synthesis of BDNF, CREB, etc. Moreover, high intake of fat in neonates insures that short-term energy requirement are met in cases of hypoglycemia. This study is mainly based on animal models, moreover, sucrose effects may differ in humans (in infants lactose is the main component of milk and is not associated with the typical analgesic effect demonstrated by sucrose ingestion).

Soares et al. (44) analysed the facilitating effect of the lipids from goat milk and conjugated linoleic acid during lactation on an excitability-related phenomenon in the brain such as cortical spreading depression (CSD) in rodents, associated with migraine and epilepsy, linking maternal diet to increased susceptibility to migraine-like phenomena in offspring. Unfortunately, experimental diet models may not reflect typical human nutrition; moreover, the association with migraine-like states remains indirect.

#### 6.3.2 Evidence from human studies

Yam et al. (43), dedicated some parts of the review, analysing how free fatty acids and dietary fats influence HPA axis regulation in both rodents and humans, finding that FFAs modulate adrenal glucocorticoid production differently in rodents (stimulation) and humans (inhibition). But human evidence remains largely correlative and based on biochemical observations rather than controlled dietary trials.

Galland (45) reviewed the role of intestinal microbiota integrity in pain and migraine, suggesting that pro-inflammatory substances related to increased intestinal permeability may reach the trigeminovascular system and trigger migraine-like attacks in human, highlighting diet-microbiota interactions. Other studies suggested a link between migraine and various inflammatory diseases, such gastrointestinal disorders, or increased intestinal permeability (46, 47). As a narrative review, Walker’s study does not establish causality, moreover, data on dietary interventions remain limited and heterogeneous.

## 7 Long-term implications

Children exposed to suboptimal prenatal or postnatal nutrition may exhibit:

- Increased baseline cortisol levels (a biomarker of chronic stress).
- Greater risk of developing anxiety, depression, or functional pain disorders such as irritable bowel syndrome and fibromyalgia.
- Altered pain thresholds and decreased effectiveness of endogenous pain control mechanisms.

These outcomes underscore the importance of targeted nutritional interventions during pregnancy and infancy to support optimal brain development and physiological.

## 8 Conclusion

Prenatal and postnatal nutrition are essential not only for physical growth but also for the development of neural systems governing pain and stress responses.

Potentially, all nutritional factors leading to neuroinflammation, epigenetic modifications, alterations of the hypothalamic–pituitary–adrenal axis, and of the microbiota, can determine modifications affecting the pain modulation and stress response systems.

Public health strategies regarding adequate maternal and early-childhood nutrition could reduce the burden of chronic pain and stress-related disorders across the lifespan. Unfortunately, few data have investigated this topic, so future perspectives may include more studies regarding these mechanisms, in order to understand how to improve or avoid food-correlated alterations of pain modulation and stress response, through maternal and early nutrition, identifying potential therapeutic targets, such as dietary interventions, probiotics, and SCFA supplementation.
